# CIP2A expression is increased in prostate cancer

**DOI:** 10.1186/1756-9966-29-136

**Published:** 2010-10-21

**Authors:** Markku H Vaarala, Marja-Riitta Väisänen, Ari Ristimäki

**Affiliations:** 1 Department of Surgery, Oulu University Hospital, PO Box 21, 90029 OYS, Finland; 2 Department of Pathology, Oulu University Hospital and University of Oulu, PO Box 50, 90029 OYS, Finland; 3 Department of Pathology, HUSLAB and Haartman Institute, and Genome-Scale Biology Research Program, PO Box 63, 00014 University of Helsinki, Finland

## Abstract

**Background:**

The CIP2A protein is a recently characterized oncoprotein which inhibits protein phosphatase 2A activity. Expression of CIP2A has been detected in several carcinomas, but its expression and significance in prostate cancer has not been examined so far.

**Methods:**

Expression of the CIP2A protein was studied using immunohistochemistry in prostate cancer (n = 59) and in benign prostatic hyperplasia (n = 20) specimens. The CIP2A staining scores were compared with several clinicopathological parameters.

**Results:**

Expression of CIP2A was increased in prostate cancer epithelium as compared with the benign hyperplastic epithelium (p < 0.001). The expression of CIP2A was associated with high Gleason scores (p < 0.001) and among patients treated with radical prostatectomy, CIP2A expression was associated with pre-treatment risk stratification (p = 0.011) and pathological T-class (p = 0.031). No statistically significant association was detected between CIP2A expression and prostate specific antigen concentrations.

**Conclusions:**

Expression of the CIP2A protein is increased in prostate cancer specimens and its expression is associated with poorly differentiated and high-risk tumors.

## Background

Serine/threonine protein phosphatase 2A (PP2A) is a tumor suppressor that plays an integral role in the regulation of a number of major signaling pathways which can contribute to carcinogenesis [1]. The cellular inhibitor of PP2A, named CIP2A (and also known as KIAA1524 and p90 tumor-associated antigen), is a recently identified human oncoprotein which promotes MYC protein stability by inhibiting PP2A-mediated dephosphorylation of MYC [2]. An increased expression of CIP2A has been detected in gastric [3,4], breast [5] and colon adenocarcinomas and in head and neck squamous cell carcinomas [2]. Interestingly, auto-antibodies against CIP2A were detected in over 30% of sera from prostate adenocarcinoma patients while only 1.5% of benign prostatic hyperplasia (BPH) patients were found to be positive for these antibodies [6].

The aim of this study was to investigate expression of the CIP2A protein in prostate cancer specimens and in BPH samples, and to examine whether CIP2A immunopositivity is associated with clinicopathological parameters in these patients.

## Methods

### Patient samples

Archived prostate specimens were initially collected from patients that underwent prostatectomy or transurethral resection of prostate as the treatment for prostate cancer or BPH at the Oulu University Hospital. The material consisted of 59 prostate cancer and 20 BPH tissue specimens, including radical prostatectomy specimens from cancer patients (n = 31), occult carcinomas diagnosed after transurethral resection for symptoms of BPH (n = 13) and palliative transurethral resections from prostate cancer patients who underwent castration therapy (n = 15). There were 1, 13, 7, 15, 1, 13 and 9 prostate tumors with Gleason scores of 4, 5, 6, 7, 8, 9 and 10, respectively. Information about corresponding Gleason scores, disease stages and prostate-specific antigen (PSA)-concentrations preceding tissue sampling were obtained from patient records. The Ethics Council of The Northern Ostrobothnia Hospital District approved the research plan.

### Immunohistochemistry

Paraffin-embedded blocks were cut into sections of 4 μm in thickness and mounted on pre-coated slides. The sections were then deparaffinized in xylene and rehydrated in a descending ethanol series. In order to enhance immunoreactivity, the sections were incubated in TRIS-EDTA, pH 9.0, and boiled for 15 min. Endogenous peroxidase activity was eliminated by incubation in hydrogen peroxide and absolute methanol. The antibody used in the study was a rabbit polyclonal antibody agains human CIP2A (NB100-74663, Novus Biologicals, Littleton, CO, USA, dilution 1:400). The bound antibodies were visualized using the Envision Detection System (K500711; Dako Denmark A/S), and DAB (diaminobenzidine) was used as a chromogen. Omission of the primary antibody served as a negative control.

### Scoring

The immunopositivity of CIP2A was graded in each sample based on the intensity of the cytoplasmic immunoreactivity in the cancer cells: 3 was strong, 2 moderate, 1 weak, and 0 negative. Using these criteria, the immunostaining results were evaluated independently by two observers (MRV and MV). Interobserver correlation was calculated from the independent evaluations. For cases with discrepancy, a consensus was reached during a common evaluation session.

### Statistical analyses

Between group comparisons were performed using Fisher's exact test for categorical variables. Continuous variables were compared with CIP2A staining using the Student's t-test or the Mann-Whitney U-test. The intraclass correlation coefficient (ICC) was calculated for the two evaluators of CIP2A immunostaining. Two-tailed p-values are presented and SPSS for Windows 15 (Chicago, IL, USA) was used for statistical analyses.

## Results

### CIP2A expression is increased in prostate cancer

**Figure 1:**
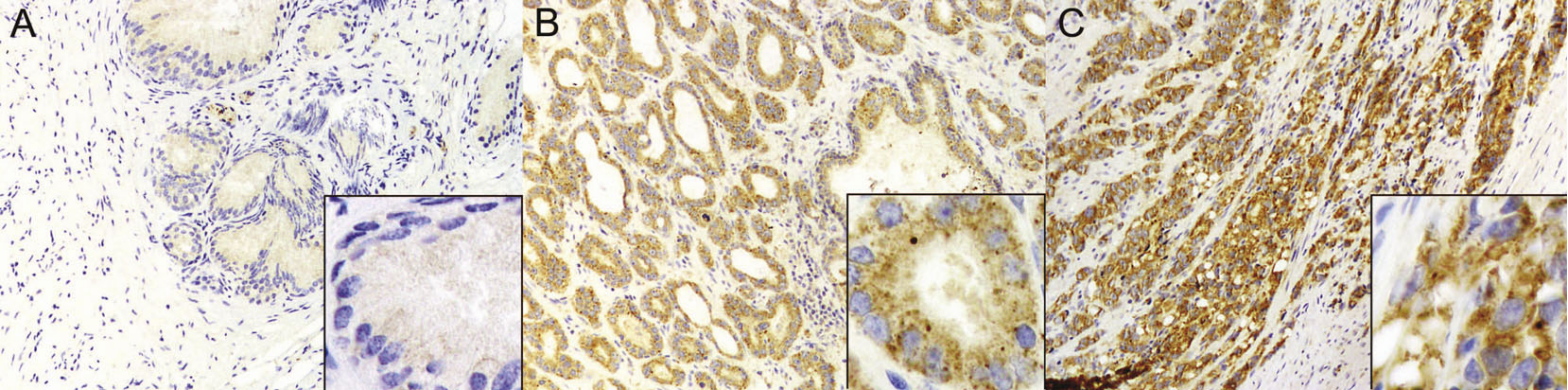
**Expression of CIP2A in benign prostatic hyperplasia and in prostate cancer**. Immunohistochemical detection of CIP2A protein expression in benign prostatic hyperplasia specimens (A) and in prostate cancer specimens (B-C). The representative Gleason scores of 6 (B) and 9 (C) are presented. Diffuse, weak cytoplasmic staining of CIP2A was present in hyperplastic tissues, whereas the staining pattern in cancer cells showed coarsely granular cytoplasmic positivity. Magnification × 100, and in inserts × 400.

Expression of the CIP2A protein was studied using immunohistochemistry and archival tissue specimens of prostate adenocarcinoma (n = 59) and BPH (n = 20). The ICC was calculated for the two evaluators of CIP2A, was and was found to be at an acceptable level (ICC = 0.93, 95% confidence interval 0.89 to 0.96). The clinical characteristics of the prostate cancer patients are presented in Table 1. All except for two prostate cancer specimens (96.6%) exhibited CIP2A immunopositivity in the prostate adenocarcinoma cells, but no staining was present in the stroma. Staining of CIP2A was also detected in epithelial cells of the hyperplastic epithelium (Figure 1). However, while in most cancer specimens (73%) the staining pattern was a coarse granular cytoplasmic positivity of moderate or strong intensity, the hyperplastic samples only stained weakly in an almost uniform manner (90%). For further analysis, CIP2A immunopositivity was divided into negative (score 0-1) vs. positive (scores 2-3) subgroups. The staining scores in the benign and malignant prostate specimens are presented in Table 2, which shows that CIP2A expression was significantly higher in prostate cancer specimens than in hyperplastic specimens (p < 0.001). In conclusion, these results suggest that expression of the CIP2A protein is increased in the epithelial cell compartments of prostatic adenocarcinoma.

**Table 1:** Clinical characteristics of the prostate cancer patients

Gleason score	n (%)
4-6	21 (35.6)
7	15 (25.4)
8-10	23 (39.0)
**PSA (ng/ml)**	**mean (SD)**

Radical prostatectomy patients (n = 31)	9.1 (5.0)
Other prostate cancer patients (n = 28)	59 (169)
**Preoperative risk group**	**n (%)**

Low-risk group (cT1a-cT2a, N0, M0 and Gleason score ≤6 and PSA <10 ng/mL)	7 (22.6)
Intermediate-risk group (cT2b or PSA 10-20 ng/mL or Gleason score 7)	16 (51.6)
High-risk group (cT2c or higher or Gleason score >7 or PSA >20 ng/mL)	8 (25.8)

**Table 2:** CIP2A immunostaining intensity in benign prostatic hyperplasia and prostate cancer.

		CIP2A immunostaining	
	n	negative	positive
Hyperplasia	20	18 (90.0%)	2 (10.0%)
Prostate cancer	59	16 (27.1%)	43 (72.9%)

p < 0.001 (Fisher's exact test)

### CIP2A expression is increased in aggressive prostate tumors

The staining intensity of CIP2A increased with increasing Gleason score, as the mean Gleason scores for CIP2A-negative and positive tumors were 5.5 and 8.0, respectively (p < 0.001). When the tumor specimens were stratified according to their clinically relevant Gleason scores as low risk and high risk tumors, there were significantly more CIP2A-positive cases among tumors with Gleason scores of 7-10 compared to those with Gleason scores of 6 or less (Table 3; p < 0.001). We further evaluated the association between CIP2A staining and pre-treatment clinical prostate cancer risk group stratification based on PSA values, Gleason scores and clinical tumor staging [7] among patients treated by radical prostatectomy (n = 31). There were 2 (28.6%), 10 (62.5%) and 8 (100%) CIP2A-positive tumors among the low, intermediate and high-risk tumors, respectively. These data show that CIP2A expression was less frequent in low-risk tumors than in high-risk tumors categorized by the pre-treatment risk stratification (p = 0.011). Furthermore, pathological T-class had a positive association with CIP2A staining intensity, as the proportion of CIP2A-positive tumors was larger among locally advanced disease samples compared to organ confined disease samples (p = 0.031). The PSA value alone and CIP2A staining intensity did not show any association (p = 0.13). There were 6 and 3 patients with biochemical or clinical progression after radical prostatectomy, with follow-up times of 3-77 and 2-41 months, respectively. Only one patient who had radical prostatectomy died of prostate cancer. The low number of patients with a progressive disease did not enable us to evaluate the prognostic role of CIP2A expression in this material. Taken altogether, CIP2A staining intensity increased significantly with increasing Gleason score, increasing pre-treatment clinical risk group stratification and increasing pathological T-class after radical prostatectomy, which are all associated with aggressive behavior of prostate cancer.

**Table 3:** CIP2A immunostaining intensity in low and high Gleason score tumors.

		CIP2A immunostaining	
	n	negative	positive
Gleason score 4-6	21	14 (66.7%)	7 (33.3%)
Gleason score 7-10	38	2 (5.3%)	36 (94.7%)

p < 0.001 (Fisher's exact test)

## Discussion

In the present study we demonstrated an increased expression of CIP2A in the human prostate cancer epithelium as compared with BPH. Furthermore, when the tumors were stratified according to the Gleason score, increased CIP2A expression was detected in the subgroup of high Gleason scores (grades 7-10) when compared to the lower Gleason scores (grades 6 or below). In addition, we demonstrated a positive association between prostate cancer preoperative risk stratification and CIP2A expression, further supporting the potential prognostic significance of CIP2A in prostate cancer. The prognostic significance of CIP2A in prostate cancer needs to be evaluated in a larger cohort with sufficient follow-up times.

The CIP2A protein is expressed in human gastric cancer [3,4,8], and it promotes proliferation of gastric cancer cells [3,4]. It has been assumed that CIP2A facilitates cell proliferation at least in part by promoting MYC stability. Furthermore, CIP2A has prognostic significance in certain subgroups of gastric cancer [4]. The CIP2A protein also promoted growth of breast cancer xenografts, and expression of the transcript was found to correlate with the expression of proliferation markers and p53 mutations, and with lymph node positivity in clinical breast cancer specimens [5]. In gastric cancer cell lines, induction of CIP2A expression following *Helicobacter pylori *infection was dependent on Src and Ras/mitogen-activated protein kinase kinase/extracellular signal-regulated kinase pathways [9]. These pathways are known to play a role in the acquisition of androgen independence [10,11], which is the central therapeutic problem in the treatment of metastasized prostate cancer.

Novel serological markers are required for the diagnosis of prostate cancer, and more importantly, to diagnose potentially lethal forms of the disease. Auto-antibodies against CIP2A were detected in 13%, 5% and 3% of the sera of hepatocellular, gastric and esophageal carcinomas, respectively [8]. Subsequently, similar auto-antibodies were detected more frequently (30%) in the sera of prostate cancer patients, while only rarely (1.5%) in BPH patients. Furthermore, CIP2A auto-antibodies were present more frequently (29% vs. 16%) in the sera of prostate cancer patients with a high Gleason score (seven or higher) when compared to patients with less aggressive disease [6]. These data with the present results suggest that evaluation of CIP2A and/or the auto-antibody concentrations against it may help in the identification of aggressive prostate cancer.

We studied CIP2A expression by immunohistochemistry only, which is a limitation of the present study. However, immunohistochemistry detects the expression of the functional gene product, CIP2A protein, and measuring RNA expression levels are not always congruent with those of the protein. To this end, since our results show significant association of CIP2A protein expression with relevant clinicopathological variables, our data are important and suggest a novel link between the oncogenic CIP2A and carcinogenesis of the prostate.

## Conclusions

We showed that expression of the CIP2A protein is increased in aggressive forms of prostate cancer. Further studies are required to demonstrate the prognostic role of CIP2A in prostate cancer and its value in the identification of aggressive disease forms.

## Competing interests

The authors declare that they have no competing interests.

## Authors' contributions

MHV and M-RV evaluated the immunostainings. MHV performed the statistical analysis. MHV and AR drafted the manuscript. All authors read and approved the final manuscript..
